# Hospitals and Laboratories on Paper-Based Sensors: A Mini Review

**DOI:** 10.3390/s21185998

**Published:** 2021-09-07

**Authors:** Huaizu Zhang, Chengbin Xia, Guangfu Feng, Jun Fang

**Affiliations:** School of Bioscience and Biotechnology, Hunan Agricultural University, Changsha 410128, China; HuaizuZhang1996@stu.hunau.edu.cn (H.Z.); xiachengbin@stu.hunau.edu.cn (C.X.); GuangfuFeng@hunau.edu.cn (G.F.)

**Keywords:** paper-based sensor, lateral flow test strips, microfluidics, bioassay trace

## Abstract

With characters of low cost, portability, easy disposal, and high accuracy, as well as bulky reduced laboratory equipment, paper-based sensors are getting increasing attention for reliable indoor/outdoor onsite detection with nonexpert operation. They have become powerful analysis tools in trace detection with ultra-low detection limits and extremely high accuracy, resulting in their great popularity in medical detection, environmental inspection, and other applications. Herein, we summarize and generalize the recently reported paper-based sensors based on their application for mechanics, biomolecules, food safety, and environmental inspection. Based on the biological, physical, and chemical analytes-sensitive electrical or optical signals, extensive detections of a large number of factors such as humidity, pressure, nucleic acid, protein, sugar, biomarkers, metal ions, and organic/inorganic chemical substances have been reported via paper-based sensors. Challenges faced by the current paper-based sensors from the fundamental problems and practical applications are subsequently analyzed; thus, the future directions of paper-based sensors are specified for their rapid handheld testing.

## 1. Introduction

Indirect sensing is the essential means for humans to study microscopic matters invisible to the naked eye. From the traditional structural (resistance strain sensors) and solid-type (thermocouple and Hall sensors) sensors to the current integrated sensors, their application range, accuracy, and portability have been greatly improved [1]. The integrated sensors have occupied the mainstream of the current sensor market due to the merits of versatility, high sensitivity, and high accuracy. However, most integrated sensors have complicated preparation processes and high costs. Moreover, many of them rely heavily on the complex system with multiple components to execute sensing functions [2].

**Figure 1:**
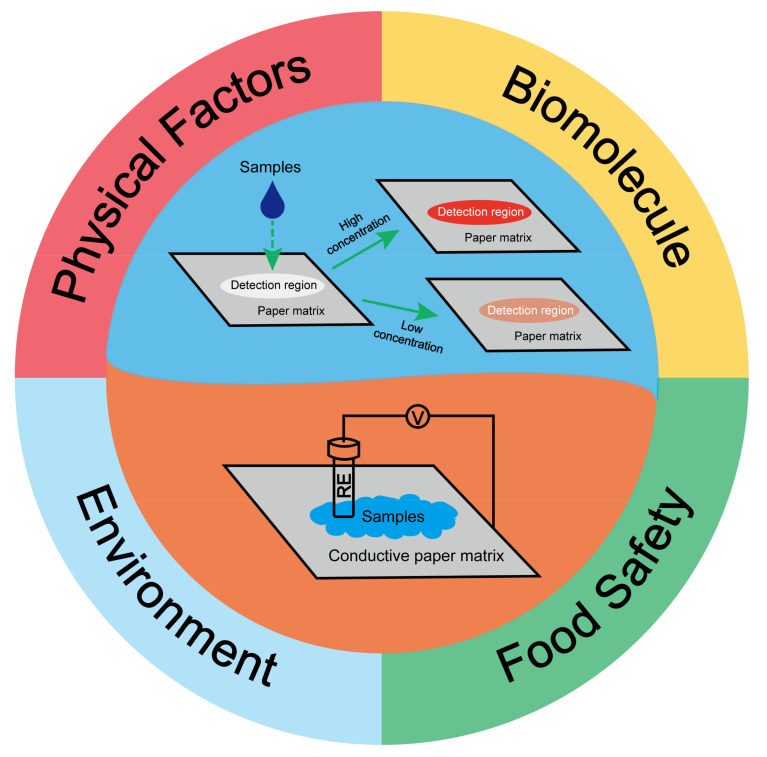
Paper-based sensors for physical factors, biomolecule, food safety, and environment detection based on the optical or electrical signal readouts.

With the popularization of biological applications, more convenient sensors are demanded to provide instrument-independent onsite detection. Paper-based sensors have the advantages of a versatile reagent-carrying ability, portability, easy operation, fast response, and low cost. This makes them strong candidates for real-time and commercial chemical or biological sensing [3,4]. Recent reports have clarified their practicability in biomarker monitoring, such as the detection of sugar [5], nucleic acids [6], phenols [7], and vitamins [8]. They have a comprehensive substance detection range and reliable high accuracy due to versatile reagent carrying, thus making them the fast-handled ‘hospitals’ and ‘laboratories’ for physical, chemical, and biological analytes sensing, even with nonexpert operation. According to the sample flowing ways on the device, it can be divided into two classifications: lateral flow assays (LFA) and microfluidic analytical devices (μ-PAD) [9]. Both the LFA and μ-PAD paper-based sensors can inspect analytes for physical factors, biomolecules, food safety, and environment detection with optical or electronic signal readouts (Figure 1). Among them, LFA paper-based sensors allow the sample to flow through the paper strip in a controlled manner during detection, while the μ-PAD ones perform in situ sensing through integrating substrate-susceptible coatings in the hydrophilic area [10]. In addition to the nonspecific assays, the paper-based sensor has substrate-specific detection with significantly enhanced sensitivity up to fM. Detection selectivity is also developed by introducing fixed antibodies or enzymes in the sensing platform [4,11]. For instance, Horseradish Peroxidase (HRP) is widely adopted to amplify paper-based sensor electrochemical or optical signals to ensure an improved selectivity and sensitivity. In the electrical sensing strategy, HRP catalyzes hydrogen peroxide to produce radicals and, thus, generate an electrochemical signal [12]. Moreover, optical signal-based analyte detection can be further realized by introducing a color-developing substrate, in which hydroxyl radicals oxidize the substrate to provide a color change of the sensor [13].

Despite specific-responding and high-accuracy substrate detection, the shelf life of paper-based sensors deserves more attention. Recently, antibody or enzyme-based immunotype methods are developed with a high accuracy and low detection limit [14]. However, their poor stability results in a short shelf life of a few days, which dramatically limits their practical applications [15]. For this, paper-based sensors with the integration of nanomaterials and even microorganisms are developed to guarantee longer shelf times, higher stability, and better environmental tolerance [16,17].

In this review, we systematically elaborated the applications of paper-based sensors in the fields of physical, biological, and chemical sensing based on the classification of detected substances (Table 1). The in-depth comprehension of the paper-based sensor constructions, operating principle, and responding mechanism are delivered subsequently. We present the recent research advances of paper-based sensors with selected representative reports in detecting humidity, pressure, biomarkers, and environmental factors. Most of them are research reported in the past five years that can provide readers with the latest understandings of the frontiers and trends of paper-based sensors. The significant potential in the reliable fast-handled onsite detection of paper-based sensors for physical, biological, dietary, and environmental factors assessment is then presented. At last, we suggestively point out the future developing directions of paper-based sensors based on the careful and holistic analysis of the challenges faced by the current paper-based sensors from the fundamental problems and practical applications.

## 2. Paper-Based Sensors for Physical Factors Sensing

### 2.1. Humidity Sensing

**Figure 2:**
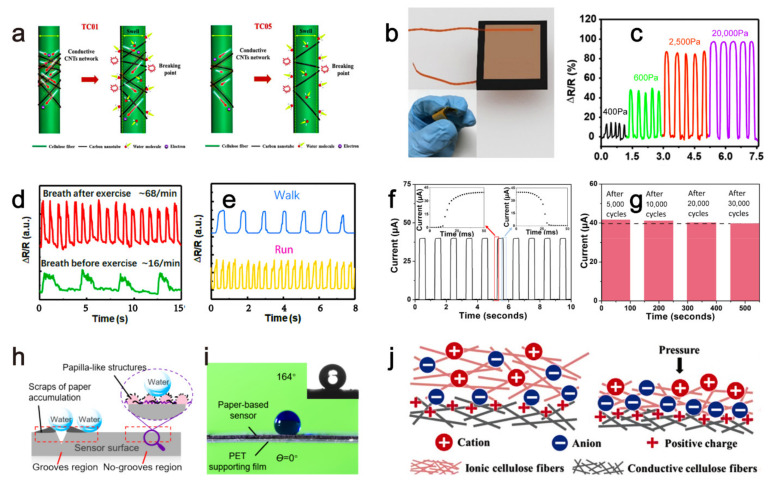
Paper-based sensor for physical factors sensing. (**a**) Microstructure change of carbon nanotubes caused by the cellulose swelling of water absorption. Reprinted/Adapted with permission from Ref. [18]. Copyright 2020 Elsevier B.V. (**b**) The GO-based pressure sensor. (**c**) Resistance changes of the GO sensor to different pressures. Reprinted/Adapted with permission from Ref. [19]. Copyright 2017 American Chemical Society. (**d**) The GO sensor for breath and (**e**) movement detection. (**f**) A 20-ms response time and (**g**) 4.6% signal loss after 30,000 cycles of folding of the strain sensor. Reprinted/Adapted with permission from Ref. [45]. Copyright 2019 American Chemical Society. (**h**) Lotus leaf bionics superhydrophobic paper-based sensors. (**i**) Superhydrophobic ability of the bionics sensor. Reprinted/Adapted with permission from Ref. [22]. Copyright 2021 American Chemical Society. (**j**) Pressure–response mechanism of the ISP pressure sensing paper. Reprinted/Adapted with permission from Ref. [23]. Copyright 2019 WILEY-VCH.

Due to the excellent hydrophilicity, chemical stability, biodegradability, abundant sources, and low price of cellulose, it was often used to prepare paper-based humidity sensors [37,38]. It was reported to utilize ways of encapsulation, blending, and coating conductive carbon nanomaterials to a cellulose matrix to prepare humidity sensors with different structures [39,40]. Although they had good detection performances, their complicated preparation process limited their practical application in nonexpert synthesis. A conductive paper-based sensor formed by self-aggregation based on electrostatic mutual attraction significantly reduced and simplified the cost and manufacturing process [18]. In this strategy, the surface of the cellulose fiber was negatively charged after 2,2,6,6-tetramethylpiperidine-1-oxyl oxidation treatment. In contrast, the carbon nanotubes were positively charged after the cationic surfactant (cetyl trimethyl ammonium bromide) treatment. Then, the negatively and positively charged cellulose fibers and carbon nanotubes were mixed to allow adsorption of the carbon nanotubes to the cellulose surface to form conductive fibers, which further constructed the paper-based sensors. A large number of hydrophilic hydroxyl groups on the cellulose fiber surface endowed it with an excellent water vapor adsorption performance and promoted the transfer of electrons from water molecules to carbon nanotubes. During the detection, cellulose swelling after water absorption would disturb the sensor’s conductive property due to the conductive carbon nanotube network damage, thereby realizing humidity detection based on an electrical signal variation (Figure 2a). Based on the conductive network structure formed by carbon nanotubes, the sensor had extremely high sensitivity (87.0%), an extremely low response limit (100%), wide response range (11–95% RH), excellent linearity (R^2^ = 0.995), good bending (curvature up to 2.1 cm^−1^) and folding (more than 50 times) durability, and excellent stability (more than 3-months exposure to air). It had potential application in fields such as breathing monitoring, air humidity monitoring, and non-contact switches.

### 2.2. Mechanical Sensing

In addition to being used for humidity sensors, the good anti-extrusion property of the paper matrix also endows it with a superb advantage for constructing strain sensors. Many paper-based sensors were designed for the handheld detection of strain pressure based on the change of the resistance, capacitance, and triboelectric current signal [41]. Flexible pressure sensors attracted gradually increasing attention owing to their great potential applications in wearable devices and human–computer interaction interfaces [42]. Especially in practical detections, people have urgent demands for pressure sensors with high sensitivity, a wide measurement range, and low costs. However, traditional electronic pressure sensors failed to achieve detection with a high sensitivity and extensive working range in a flexible being way due to the equipments’ rigidity, resulting in their limited applications in the wearable field. Since graphene material was discovered in 2004 [43], it had been widely used in flexible mechanical devices. The graphene-based mechanical sensor shows a high detection sensitivity in a small pressure range due to its unique electrical and mechanical properties. However, there are shortcomings and problems, such as a complex preparation process, poor repeatability and consistency, and low yield for the production of graphene [44]. In addition, graphene paper-based pressure sensors with high sensitivity in a large measurement range were rarely reported. There is an urgent need to develop a new type of pressure sensor to achieve breakthroughs in both the sensitivity and working range. Recently, researchers proposed a high-performance pressure sensor by utilizing the graphene-containing paper [19]. Researchers mixed tissue paper with graphene oxide (GO) solution to obtain the GO paper sensor (Figure 2b). This method significantly improved the conductivity of the paper with simple and easy synthesis and operation, which greatly improved the production potential of graphene materials. In investigating the sensitivity of the paper sensor to pressure, it was found that the relative change of the resistance of the paper-based sensor reached as high as 17.2 kPa^−1^ in a broad pressure range of 0–20 kPa (Figure 2c). This meant it could perform accurate and reliable detection under a wide range of pressures. In addition to graphene impregnation, poly(3,4-ethylenedioxythiophene) polystyrene sulfonate impregnation of the paper matrix was also feasible in constructing a paper-based pressure sensor [45]. The as-prepared sensor could accurately trace the wrist pulse, speech, breathing, and exercise statuses (Figure 2d,e), indicating its great potential in smart wearable devices for health monitoring and exercise detection, in which the conduction current varied with the change of the contact area between the porous microstructure paper layers under pressure stimuli. Subsequently, the sensor could realize the capture and feedback of external stress stimuli accompanied by a response time of 20 ms (Figure 2f). At the same time, this paper-based sensor has a slight signal loss of 4.6% after 30,000 pressure cycles, implying its excellent stability and practicality (Figure 2g).

Compared with the aforementioned contact sensing, non-contact acoustic detection is more challenging. A paper-based sensor with porous microstructure and stacked microstructure prepared by direct laser writing strategy was developed to act as an acoustic strain sensor [20]. As a strain sensor, this device could detect the amplitude and frequency of the applied strain and detect the direction of tensile and compressive deformations. Furthermore, this sensor was susceptible to weak pressure signals (such as sound) and could even distinguish the seven piano notes.

To better understand the working principle, it is genuinely a tangibility that the electrical signals of the paper-based sensors, those that adopted the spraying of conductive materials on the paper matrix, are affected by the microstructure changes between the conductive materials under high-pressure conditions. For this, high pressure would suppress the conductive materials more densely, resulting in a decreased resistance of the paper-based sensors to give a raised current with the same voltage. Additionally, the distance change between the conductive material and the electrode under a relative low-pressure range would simultaneously give a current disturbance of the paper-based sensors. An ultra-sensitive pressure sensor made of photolithography and copper deposition strategy was structured by directly spraying a uniform polyvinyl pyrrolidone (PVP) film that contained carbon nanotubes (CNT) onto the surface of the paper, which could respond to pressure in a wide measurement range (0–140 kPa) [21]. The compressed PVP film would contact the paper surface containing the interdigital electrodes, and the gaps between the interdigital electrodes were filled with conductive PVP film, allowing the sensor to form a complete electrical signal working circuit. For the working principle, a closer distance between the interdigital electrodes and PVP film or the CNT in the PVP film would lead to a decreased resistance, thus giving an increased current intensity that was measured by a semiconductor parameter analyzer. For this pressure sensor, the connection area between the PVP film and the interdigital electrode affected the electrical signal change in a small pressure range, while the internal distance change of the CNT in PVP film had a major effect on the electrical signal in the large pressure area. Furthermore, the array unit made by the pressure paper could detect the spatial pressure distribution, which was expected to have a high potential in the application of the human–computer interaction interface. This would bring new inspiration to researchers for the construction and mechanism illustration of paper-based electronic signal sensors.

The paper-based sensors mentioned above have a drawback, which is, when they are exposed to high moisture, their detection functions are usually lost. In fact, there is an increasing demand for high-performance and hydrophobic flexible paper-based sensors due to emerging applications such as human motion monitoring, sweat or humidity detection, and certain underwater tests. However, most paper-based electronic devices are prone to failure when exposed to water, implying their incompetent sensing in humid or underwater environments. For hydrophobicity mimicking, the micro-nano double-papillary structure on the lotus leaf’s surface confers it with a strong hydrophobicity, which provides researchers with an excellent example for producing hydrophobic materials on the nanoscale. Recently, the paper-based sensor showed excellent practicability in wet and underwater environments based on lotus leaf bionics with abundant papilla-like structures (Figure 2h) [22], and based on the bionic grooved silt sensillum of the scorpion’s walking leg, this sensor showed a high sensitivity towards vibration, in which the deformation of the sensor caused by the vibrations increased/decreased the distance between the two walls of those conductive grooves and subsequently increase/decrease the sensor’s resistance. The gauge factor, which represented the ratio of relative change in electrical resistance to the mechanical strain, reached a high coefficient of 263.34. A higher gauge factor meant a more sensitiveness change of the material to mechanical strain. Based on this, the sensor showed a high strain resolution of 0.098%, a short response time of 78 ms, a water contact angle of 164°, and excellent stability in 12,000 cycles (Figure 2i). It was suitable for wearable electronic devices monitoring body movements in real-time with an unaffected signal by sweat. Furthermore, it could even work in an underwater environment for the detection of subtle underwater vibrations. In comparison, another superhydrophobic paper-based sensor showed strong acid/alkali and moisture resistance based on the Hf-SiO_2_ coating, allowing high self-cleaning and corrosion resistance capabilities in strong acid/alkali solutions [46].

The aforementioned paper-based sensors were mainly developed based on pressure-sensitive resistance, capacitance, and triboelectric current signals. They had concerns like complicated preparation methods, low responding speeds, and narrow linear response ranges. In addition, the most currently reported paper-based sensors failed to make full use of the paper’s printable, cuttable, and foldable properties. Researchers proposed the ion sensor paper (ISP) sensor platform based on the flexible ion sensor mechanism [23]. In this platform, an increment of the contact area among the ionic and conductive cellulose fibers was permitted once loading an external pressure that would cause a capacitance rise of the ISP sensor (Figure 2j). Based on the pressure-dependent capacitance change, the external pressure was measured. The sensor could be structured in the form of a two-dimensional plane or three-dimensional origami by specific operations of the ISP (such as printing, cutting, folding, or gluing), indicating its direct printability, custom cutability, and three-dimensional foldability. In addition, the sensitivity of the ISP device reached 10 nF/kPa/cm^2^, which was several thousand times of the capacitive–current paper sensors. Moreover, it had a fast mechanical response time of one millisecond and high response linearity with a resolution of 6.25 Pa. This work showed the proof of concept of a flexible and intelligent electronic sensing paper which had a significant sensing advantage and application potential in human-machine interface, smart packaging, and wearable healthcare products.

## 3. Paper-Based Sensor for Biomolecule Sensing

The development of biomolecular detection technology is the focus of contemporary molecular biology and genetics research, which demands high detection accuracy and trace sample analyses. It has been widely used in medicine, food, health, and other fields [47]. However, most molecular detection technologies require sophisticated and expensive heavy instruments, limiting their practical applications. In the past few decades, paper-based sensors have shown their significant ascendancy in biomolecule detection with low cost, good portability, high accuracy, and good reproducibility. They can detect a wide range of biological molecules, such as nucleic acid, protein, sugar, antigen/antibody, vitamin, neurotransmitter, and antibiotic.

### 3.1. Nucleic Acid Sensing

Nucleic acid regulates the appearance and physiological metabolism of organisms [48]. At present, it is known that a large number of diseases are caused by DNA or RNA mutations [49]. Therefore, detecting specific nucleic acid targets benefits the early diagnosis and early treatment of related diseases, which has great clinical significance.

Malignant gene mutations usually result in cancer, and there are usually marker RNA transcriptions after cells became cancerous. The detection of those marker RNAs has high confidence in the early detection of cancers [50,51]. For instance, miRNA−21 is one of the early markers of glioma cancer. Fakhri and coworkers proposed a paper-based sensor that could recognize miRNA−21 by fixing the DNA template onto the Ag/Pt nanoclusters, which showed a linear detection range of 1–700 μM and a detection limit of 4.1 pM [13]. The principle of this sensor lay in that, after the capture of miRNA−21 by the DNA template based on the complementary-based hybridization, the quenched peroxide catalytic ability of Ag/Pt nanoclusters was recovered, thus realizing the colorimetric detection of miRNA−21 with the association of 3,3′,5,5′ tetramethylbenzidine (TMB) and hydrogen peroxide. The reason for the adoption of Ag/Pt mixture nanoclusters in this strategy lay in its significantly higher catalytic activity to peroxide hydrogen than single Ag or Pt nanoparticles. For improvement of the accuracy and avoidance of false-negative signals, Tian and coworkers designed a paper-based sensor that detected both microRNA−141 (miR−141) and microRNA−21 (miR−21), with a detection limit of 0.1 fM [46].

**Figure 3:**
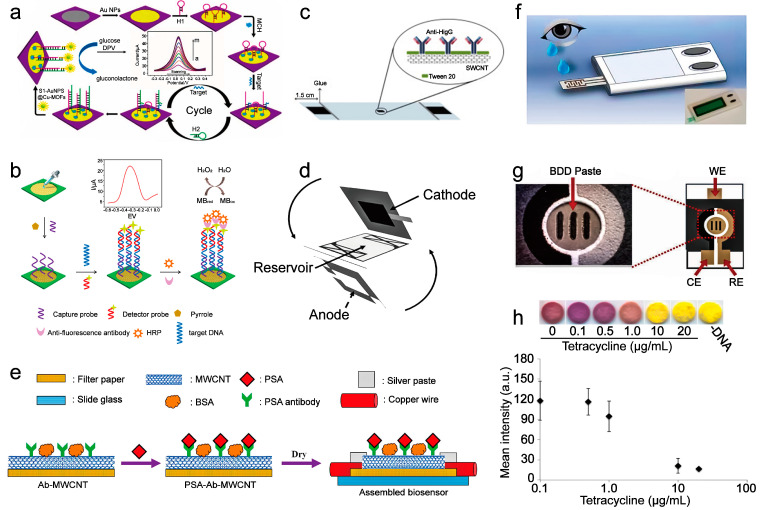
Paper-based sensor for biomolecule sensing. (**a**) Hairpin-assembly signal amplification of the miRNA−155 bio-sensor. Reprinted/Adapted with permission from Ref. [25]. Copyright 2018 Elsevier B.V. (**b**) Enzyme-based colorimetric detection of EGFR. Reprinted/Adapted with permission from Ref. [12]. Copyright 2017 Elsevier B.V. (**c**) The HIgG chemiresistor sensor with ideal attachment of the anti-HIgG onto the SWCNTs. Reprinted/Adapted with permission from Ref. [54]. Copyright 2013 Elsevier B.V. (**d**) The three-dimensional glucose sensor formed by folding the chitosan/GOx anode, air cathode, and anode reservoir together. Reprinted/Adapted with permission from Ref. [55]. Copyright 2016 Elsevier B.V. (**e**) Electrical resistance variation caused by antigen–antibody binding for prostate cancer antigen detection. Reprinted/Adapted with permission from Ref. [15]. Copyright 2018 Elsevier B.V. (**f**) Graphic illustration of the ascorbic acid measurements with the OcuCheck sensor. Reprinted/Adapted with permission from Ref. [56]. Copyright 2015 Sci Rep. (**g**) The three-electrode sensor was composed of a carbon electrode (CE), Ag/AgCl (RE), and working electrode for the norepinephrine and serotonin analyses. Reprinted/Adapted with permission from Ref. [30]. Copyright 2017 American Chemical Society. (**h**) Tetracycline inhibited β-galactosidase expression for the colorimetric detection of tetracycline with the assistance of color-developing substrates. Reprinted/Adapted with permission from Ref. [57]. Copyright 2017 The Society for Biotechnology.

As depicted above, the signal of the sensor that realized the codetection of miR−141 and miR−21 for early cancer diagnosis depended on the concentration of miR−141 and miR−21. However, there are low concentrations of the two contents in organisms, thus resulting in a weak signal of the sensor during the real sample detection. Wang and coworkers delivered a hairpin-assembly signal amplification strategy in response to this trace detection situation [25]. The application of this strategy significantly improved the sensor’s ability to detect trace substrates in real samples, in which Cu-based metal-organic frameworks were used to fix DNA strand 1 (S1), hairpin probe H1, hairpin probe H2, and AuNPs. The detection process was performed as follows: (1) miRNA−155 bonded to H1, opening the hairpin structure of H1. (2) H2 competitively combined with H1 and then released miRNA−155. (3) miRNA−155 continued to bind to other H1s and circulated this process. (4) H1–H2 duplexes were combined with S1-AuNPs@Cu-MOFs to oxidize glucose to generate an electrical signal, which showed a detection limit of 0.35 fM and a broad linear detection range (1.0 fM–10 nM) (Figure 3a).

In addition to inorganic nanoenzymes, the coupling of natural enzymes is also an effective strategy to explore paper-based sensors with an enzyme-linked assay, which can simultaneously reduce environmental pollution after the sensor is used. The enzyme-linked detection of nucleic acids based on the DNA template strategy also showed high confidence. Epidermal growth factor receptor (EGFR) is a type of receptor in the human body that interferes with the maintenance and growth of epithelial tissues, and the irregular expression of EGFR is related to the occurrence of lung cancer [52]. Detection of the mutation of the EGFR gene is beneficial for the early diagnosis of lung cancer. Recently, Tian’s team designed a paper-based DNA biosensor that detected mutations of the epidermal growth factor receptor (EGFR) with enzyme-based signal generation [12]. The sensor captured the target DNA associated with EGFR mutations through immobilized oligonucleotides. After the binding, the HRP at the end of the oligonucleotide chain would allow this sensor an apparent electronic signal with the catalysis of hydrogen peroxide (Figure 3b). The noninvasive diagnosis for EGFR mutations with the patient’s saliva was performed with a low detection limit (0.167 nM), considering that the concentration detected in the real sample was 2.455 nM. This strategy showed a relatively wide detection range of 0.5–500.0 nM, which was broad enough to detect the real sample. For another enzyme-based detection of nucleic acids, Lin’s team further developed a more convenient color test paper sensor [53]. During the detection, the sensor captured the target transcription factor, conferring it with the ability to cycle the production of the G-quadruplex sequence under the assistance of the exonuclease III and P3 hairpin sequence. After that, K^+^ and hemin were added to form DNAzymes with the G-quadruplex sequence. Then, the DNAzymes intermediated the production of aminochrome from dopamine, thus realizing a distinguishable color change of the paper from the colorless dopamine contamination to the red color aminochrome contamination.

### 3.2. Protein Sensing

Protein accounts for 19.4% of the total body weight [58], and it participates in the pivotal regulation of physiological metabolism, signal pathways, and oxygen transmission. Additionally, detecting the physiological levels of specific proteins can allow the dynamic detection of liver, cardiovascular, Parkinson’s syndrome, Alzheimer’s disease, and tumors [59,60]. Recently, the fluorescent immunoassay detection of the carcinoembryonic antigen (CEA) by a paper-based sensor has been established with a detection limit of 0.041 ng mL^−1^ [26]. In this strategy, the fluorescence enhancement triggered by the ammonia absorption of the detector was utilized to perform the fluorescence-based detection of the target antigen. Glutamate dehydrogenase (GSH) was fixed on the gold nanoparticles, and then, the complex was further modified with the antibody of CEA. When there was CEA in the solution, the GSH-contained complex would bind to the CEA. Then, the CEA would subsequently bind to the antibody that immobilized on the sensor, causing capturing of the GSH-contained complex by the paper-based sensor. The GSH in the complex would then catalyze sodium glutamate in the solution to produce NH^4+^. After adding sodium hydroxide, NH^4+^ would react with OH^−^ to generate ammonia gas, thus enhancing the sensor’s fluorescence. In addition, Pozuelo and coworkers developed a paper-based chemoresistance sensor that integrated single-walled carbon nanotubes and anti-human immunoglobulin G (anti-HIgG) to achieve the detection of the HIgG protein (Figure 3c) [54], in which the detection limit reached the picomolar level. Researchers also proved that the sensitivity of the sensor was tunable with the varying density of the single-walled carbon nanotubes. Based on the immunoassay philosophy, glycated albumin, one of the indicators of diabetes, was also quantitatively detected in the real blood samples to assess the diabetes progression with a paper-based sensor [61].

### 3.3. Sugar Sensing

In addition to the quantitative detection of protein, paper-based sensors for sugar detection also receives tremendous attention nowadays. Sugar is one of the most widespread organic substances in nature, and it plays a crucial role in our daily diet and health. Especially, blood sugar is the most critical health indicator of the human body relating to the operation of various tissues and organs and regulating the person’s overall physiological balance. The paper-based sensor designed by Andrade’s team for glucose detection was the most primitive, which consisted of filter paper, a sprayed platinum layer, and a nafion membrane [62]. The Nafion membrane acted as a carrier for fixing glucose oxidase, which could eliminate the interference of negatively charged species in the detection substrate. The glucose oxidase fixed on the sensor oxidized glucose with hydrogen peroxide production, thereby generating electrical signals. The detection limit was 10^−4.5^ M, and the linear detection range was 10^−4^–10^−2.5^ M.

Unlike Andrade, Fischer designed a paper-based glucose sensor with an enzyme fuel cell model [55]. The three-dimensional sensor structure was formed by folding the chitosan/GOx anode, air cathode, and anode reservoir together (Figure 3d). Trace amounts of substrates (less than 20 µL) were enough for the detection use, which showed a sensitivity of 0.02 µA mM^−1^ and could maintain a high accuracy in an extensive concentration range (1.0–5.0 mM). Unlike the traditional electrical-signal sensor, this sensor formed a current path between the anode and the cathode through enzyme-mediated glucose oxidation, allowing the sensor’s independence from external power during the detection.

Based on the idea of an integrated sensing platform like the abovementioned works, Calabria’s group developed a paper-based sensor system for detecting L-lactate [27]. The system consisted of polyelectrolyte with coupled enzyme and a smartphone-based device. The former performed a cascade oxidation reaction of lactate and catalysis of peroxide hydrogen to achieve color development of the colorless substrate miRNA−155. The latter was adopted to distinguish the hue saturation value of the sensing paper, then accurately calculate the color change of the sensor. Compared with human eye recognition, the sensitivity of this sensor was greatly improved. When detecting lactate, the detection limit of the system could reach 0.1 mM L^−1^, which was significantly lower compared to the salivary concentration of the anaerobic threshold (2 mM L^−1^). Yet, it should be noted that 1 min of time was needed to present a stabilized color of the paper sensor due to its dynamic enzyme reaction. For another integrated glucose-sensing platform, Leonor’s group prepared a multilayer paper-based sensor for glucose sensing. The device consisted of paper, a platinum layer, Nafion layer, and GOx/chitosan/PVA(D) enzyme membrane layer [28]. This sensor presented the innovation of greatly improved stability (more than one month at 4 °C) of GOx by utilizing polyvinyl alcohol for enzyme immobilization and greatly enhanced the catalytic efficiency by introducing chitosan for the even dispersion of GOx. At the same time, the platinum layer was used as the working electrode to allow a sensor sensitivity of 0.03–1.0 mM and a detection limit of 0.02 mM.

To further realize glucose detection in the human body, traditional methods inevitably cause damage to the detection host due to the requirement of blood samples. Recently, Huang and coworkers developed a noninvasive paper-based silver@gold nanoprism sensor for glucose detection [63]. The sensor was composed of silver@gold nanoprisms, Ir-Zne-MOFs, and glucose oxidase. A wide linear working range of 0.05–30 mM, fast responding speed of 0.5 s, and low detection limit of 0.038 mM made this strategy superior to the other paper-based sensors dedicated to glucose detection. During detection, the greatly enhanced emission intensity of the sensor’s phosphorescence (55 times) by silver@gold nanoprisms was responsible for its high sensitivity and low detection limit. Additionally, in the detection of human urine samples, this sensor showed good stability and accuracy.

### 3.4. Antigen/Antibody Sensing

Antigen/antibody is one of the crucial indicators for detecting the physiological conditions of the human body. Based on antigen/antibody immune hybridization, researchers can quickly detect viruses, tumor cells, and other physiological abnormal factors. The most typical and essential strategy in the design philosophy of antibody/antigen sensors is to capture the analyte using antigen/antibody precisely and follow an amplified detection signal. The specific binding reaction of the antigen to the antibody is a practical principle for the accurate detection of cancer antigen markers. For instance, Saurabh’s group realized the detection of the cancer biomarker carcinoembryonic antigen with an antibody-based paper sensor [14]. Ji’s group developed a paper-based sensor for the sensitive and customized detection of the prostate cancer antigen [15]. The sensor realized the detection of the prostate-specific antigen based on the principle of immunodetection (Figure 3e). During the sensor construction, the specific antibodies of the prostate antigen were introduced and activated on multiwalled carbon nanotubes to allow antigen capture. Then, the immune reaction would trigger a resistance change of the paper sensor that is detectable with a desktop digital multimeter, thus executing antigen sensing. Though the detection limit of the sensor was 1.18 ng/mL, the short shelf life (2 h) needed to be improved. At the same time, researchers also developed a paper-based sensor for the simultaneous detection of the carcinoembryonic antigen and HIV antigen [29]. In that model, the recognition between β-cyclodextrin-coated gold nanoparticles and 1-adamantane acetic acid or tetrakis (4-carboxyphenyl) porphyrin was employed to assist the layer-by-layer self-assembly reaction, which amplified the final optical signal after several rounds of reaction. Due to the amplified design concept, this platform showed a broad dynamic detection range of the analyte concentration of seven orders of magnitude, which effectively covered the needs of the clinical ranges. The customized detection of carcinoembryonic antigen and HIV-1 capsid p24 antigen in actual serum samples could be performed even without dilution of the original sample.

### 3.5. Vitamin Sensing

Unlike antigen/antibody detection based on antigen–antibody recognition, paper-based sensors generally utilize nanomaterial-mediated color changes, anodization, or ascorbate oxidase coupling strategies to detect ascorbic acid. Electrical-, colorimetrical-, and enzyme-based vitamin detection by paper-based sensors was reported with a certain practicability. By utilizing the reaction between Ag nanoparticles and ascorbic acid (AA), the growth of Ag nanoparticles to clusters was realized. Subsequently, the colorimetric detection of ascorbic acid was performed with a significant color change of the sensor paper from light yellow to grey due to the cluster formation [8]. In addition, the anodic oxidation peak of AA measured by a carbon electrode could also be used to sense the AA concentration through the AA-dependent electrical signals [64]. For examination of the eye, limited training, high cost, and low equipment mobility of the traditional assays resulted in inaccuracies of the testing results and was concerned with severe ocular injuries. The state-of-the-art biosensor designed for ascorbic acid measurement in ocular fluid showed high safety, practicality, and accuracy, with significantly simplified operation steps and improved portability (Figure 3f) [56]. During the detection, ascorbic acid was dehydrogenated firstly by ascorbate oxidase. Then, the electrons were transmitted to the electronic pathway composed of graphene, thus generating an electrical signal that was positively correlated with the concentration of ascorbic acid. The sensor’s sensitivity, accuracy, and specificity were nearly twice as high as the traditional gold standard colorimetric method, which significantly improved the nursing safety of clinical eye care.

### 3.6. Neurotransmitter Sensing

Neurotransmitters, which distribute widely in tissues and organs, are essential biochemical molecules regulating the physiological functions of the central and peripheral nervous systems. Recently, Nantaphol and coworkers realized the detection of norepinephrine and serotonin with boron-doped diamonds (Figure 3g) [30]. The protocol platform could simultaneously detect norepinephrine and serotonin in wide concentration ranges with high sensitivities, for which electrode fouling caused by norepinephrine or serotonin led to a decrease of the oxidation current and shift of the positive peak potential. A reduced graphene oxide modification of the electrode was introduced to allow the sensor a better discrimination ability for the two analytes. By analyzing the cyclic voltammetry curve of the sensor, the concentration of the two substrates could be measured. The sensitivities of the current to the analyte concentration were 0.030 and 0.069 µA/µM for norepinephrine and serotonin, which showed an improved sensitivity compared with the previous work [65]. Furthermore, the anodic treatment of the electrode after detection was adopted for electrode cleaning and recycling.

### 3.7. Antibiotic Sensing

Antibiotics refer to a class of secondary metabolites produced by microorganisms (bacteria, fungi, and actinomycetes) or higher animals and plants with antipathogen functions, which interfere with other environmental organisms’ normal metabolisms and growth. It has been widely used in a wide range of fields such as agriculture, medical treatment, and animal husbandry for decades, and the followed contamination of antibiotics has received increasing attention [66,67].

Unlike the strategies that performed substrate sensing based on enzyme or inorganic materials, Abigail’s group developed a novel paper-based biosensor utilizing yeast to detect doxycycline antibiotics [16]. This bio-based paper analysis device was sensitive to detecting doxycycline in a wide concentration range (30–10,000 μg/mL), for which, in the absence of doxycycline, the expression of the reporter by gene-edited yeast was blocked. In contrast, the interaction of doxycycline with the activator would result in the biased binding of the activator to the Tet operators of the yeast to allow transcription of the reporter when in the presence of doxycycline. Though there was a good linear relationship between doxycycline (10–100 μg/mL) and the reporter, doxycycline-induced yeast death under high concentrations is also a concerning issue. The most significant merit of this live cell-based sensor was that it could maintain activity even after more than one year of storage at −20 °C. This challenged the common sense of the short shelf time for bio-based sensors and expanded the space of long-term preservation and the use of biological products. For another bio-based sensor, Duyen’s team detected antibiotics via suppression of the bacterial gene expression [57]. In which the bacteria were immobilized on the paper discs containing a colorimetric substrate, and then, the paper discs were freeze-dried to construct the sensing paper. In the absence of antibiotics, the bacteria expressed β-galactosidase. β-galactosidase hydrolyzed the colorimetric substrate to present a color change of the paper from yellow to purple. Conversely, in the presence of antibiotics, the color change was hampered due to the inhibition of β-galactosidase synthesis. Researchers proved that barrotomycin, tetracycline (Figure 3h), chloramphenicol, and erythromycin could inhibit β-galactosidase expression of the bacteria. The detection limits to the four antibiotics were 0.5, 2.1, 0.8, and 6.1 µg/mL, which was enough to detect the antibiotic content in contaminated water (20 µg/mL of oxytetracycline in the detected water sample).

## 4. Paper-Based Sensors for Food Safety Testing

**Figure 4:**
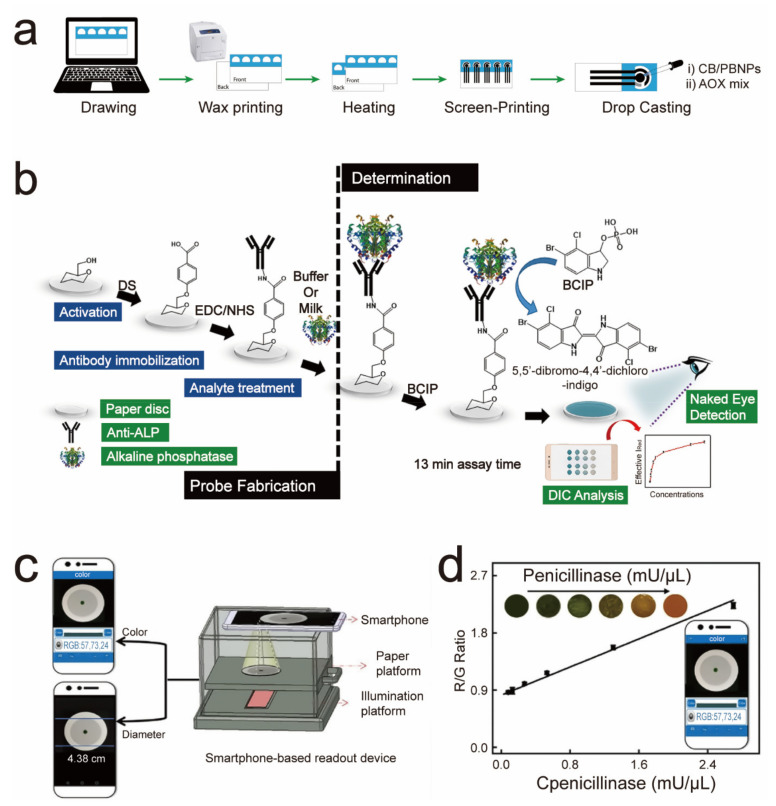
Paper-based sensors for food safety testing. (**a**) Fabrication processes of the biosensor that aimed for ethanol sensing in beer samples. Reprinted/Adapted with permission from Ref. [31]. Copyright 2017 Elsevier B.V. (**b**) Synthesis and working principles of the ALP-sensing device. Reprinted/Adapted with permission from Ref. [32]. Copyright 2019 Elsevier B.V. (**c**) Diameter- and color-based dual-signal readouts of the penicillinase sensor. (**d**) Color-based detection of penicillinase based on the pH-sensitive color change of Alg-CTAB-BTB. Reprinted/Adapted with permission from Ref. [69]. Copyright 2021 Elsevier B.V.

Except for detecting physiological molecules, paper-based sensors also receive great attention in the field of food safety testing. Paper-based sensors provide a convenient solution for food quality and safety evaluation. Recently, Cinti and coworkers developed a paper-based biosensor that allowed the detection of ethanol in beer samples (Figure 4a) [31]. The sensor was mainly composed of carbon black, Prussian blue nanoparticles, and alcohol oxidase. Among them, alcohol oxidase oxidized ethanol to produce hydrogen peroxide; carbon black assisted the production of the Prussian blue nanoparticles. Then, there was a current response due to the carbon black and Prussian blue composite reaction to peroxide hydrogen. The Ag/AgCl pseudo-reference electrode, graphite working electrode, and counter electrode were printed on paper to form a three-electrode system. This conferred the sensor’s extremely high sensitivity of 9.13 mA/mM cm^2^ compared to the previously reported 31.0 nA/mM cm^2^ [68]. Furthermore, the high sensitivity made this sensor capable of measuring ethanol with a detection limit of 0.003%vol (0.52 mM), indicating its sufficient capacity for the commercial detection of ethanol.

In milk products, the incomplete killing of pathogenic microbes will damage their palatability and quality and induce high food-borne disease risks. However, traditional protocols that evaluated the pasteurization efficiency usually took a long detection time. Recently, Mahato and coworkers developed a portable paper-based sensor for the fast and accurate onsite quantitative detection of alkaline phosphatase in the milk sample (Figure 4b) [32]. In their strategy, alkaline phosphatase (ALP), a metabolite product of dairy cow cells that mainly associated with the milk fat globule membrane in raw milk, was selected to assess whether the pasteurization was effective due to its close destruction point to the killing temperature of the pathogenic bacteria. During the detection, ALP was captured by the ALP antibody that was fixed on the paper sensor. Then, ALP catalyzed 5-bromo-4-chloro-3-indole phosphate to liberate the blue-green product. With the assist of digital colorimetric-based technology, a significant fall of the blue-green product in the actual milk sample to raw milk was observed, implying effective industry pasteurization. At the same time, Xu and coworkers designed a paper-based sensor that performed a penicillinase estimation in milk [69]. As an enzyme that widely exists in bacteria, penicillinase can specifically and efficiently catalyze and hydrolyze penicillin. A high penicillinase concentration indirectly reflects the high penicillin stress that bacteria (in milk) are subjected to. Moreover, antibiotic residues are an essential concern in the food industry today. The quantitative detection of trace amounts of penicillin and penicillinase in milk could better meet food safety and human health needs. In that hypothesis, the sensor could sense penicillinase with diameter- and color-based dual-signal readouts (Figure 4c). For color-based penicillin G detection, penicillinase catalyzed it to generate penicilloic acid, leading to the decline of the solution pH value. Then, the pH-sensitive substrate alginate-cetyltrimethylammonium-bromide-bromothymol-blue (Alg-CTAB-BTB) delivered varied colors at different pH values (Figure 4d). As for diameter-based detection, the Ca^2+^ release caused by the reaction of penicilloic acid to CaCO_3_ would induce the hydrogelation of the alginate. Subsequently, the diffuse diameters of the Alg-CTAB-BTB complex were captured with the assistance of a smartphone. For the real sample estimation, the sensor showed a high accuracy, as well as a commercial ELISA kit, indicating its highly robust and practical performance.

For more inspiration, Hidayat’s group delivered a paper-based sensor that allowed the detection of green tea polyphenols [70]. Briefly, tyrosinase fixed on the paper was used to oxidize polyphenols; after which, the redox adducts reacted with 3-methyl-2-benzothiazolinone hydrazone to generate a pink composite. For interference investigating, researchers proved the interference of less than 5% for vitamin C in the concentration range of 0.57–2.27 mM.

## 5. Paper-Based Sensors for Environmental Quality Inspection

Similar to food safety monitoring, the sensitive detection of CO_2_, phenols, and metal ions by paper-based sensors makes them handy tools for environmental quality inspections [71,72]. There is indeed a concern that most environmental inspection assays could not meet outdoor onsite detection due to the requirements of complicated instruments and a relative long detection time [73]. With properties of low cost, easy operation, great portability, and easy disposal, paper-based sensors were found reliable to detect carbon dioxide [33], airborne particulate [74], volatile organic compounds [75] in the air, heavy metals [76], phosphates [77], inorganic element ions [78,79,80], nitrite [81], and nicotine [82] in the water.

Carbon dioxide (CO_2_) plays a vital role in climate change, plant growth, and agricultural pest control. The detection of CO_2_ is an essential prerequisite for humans to reasonably adapt to the evolution of nature and a requirement to protect the environment. Wang and coworkers designed a paper-based sensor for CO_2_ detection with high accuracy based on the fluorescence assay, in which the fluorescence peak position shifted after the absorption of CO_2_ with a detection limit of 5.7 ppm [33].

**Figure 5:**
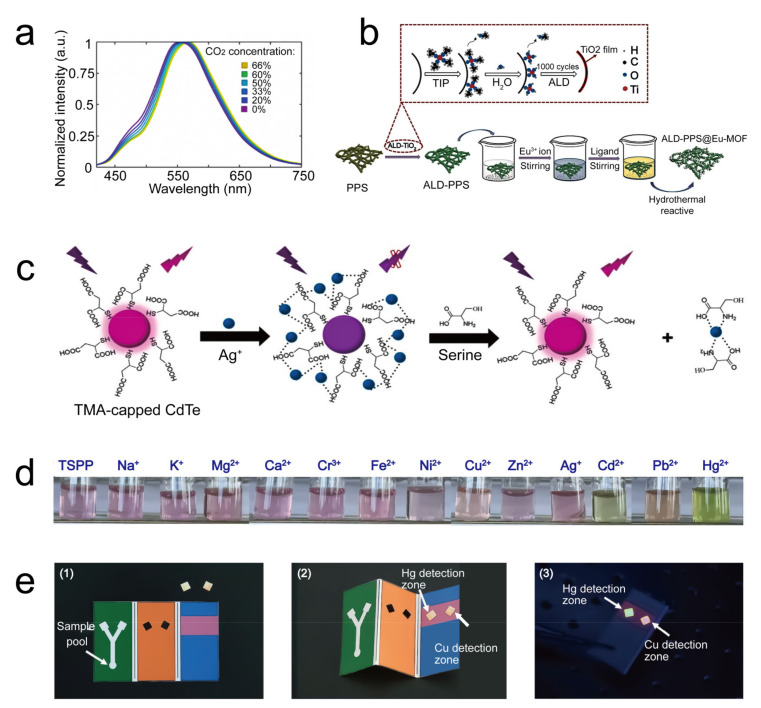
Paper-based sensors for an environmental quality inspection. (**a**) Photograph images presented the color of the phenolic biosensor with varied catechol concentrations. 1 represented the control paper, and 2 represented the working paper. Reprinted/Adapted with permission from Ref. [34]. Copyright 2020 Springer-Verlag GmbH Germany, part of Springer Nature. (**b**) Schematical representation for the synthesis of the Fe^3+^-sensitive paper-based sensor. Reprinted/Adapted with permission from Ref. [83]. Copyright 2021 Elsevier B.V. (**c**) Ag^+^ quenched and serine restored the fluorescence of thiomalic acid-modified CdTe (TMA-capped CdTe). Reprinted/Adapted with permission from Ref. [35] Copyright 2020 Elsevier B.V. (**d**) The color-developing material were used for Cu^2+^ and Hg^2+^ measurements. Reprinted/Adapted with permission from Ref. [84]. Copyright 2017 American Chemical Society and Division of Chemical Education, Inc. California, America. (**e**) Portable three-dimensional paper-based sensor for simultaneous Cu^2+^ and Hg^2+^ detection: The sample filter processing mat (**1**), detection mat (**2**), and fluorescence emission of the detection mat with the presence of Cu^2+^ and Hg^2+^ (**3**). Reprinted/Adapted with permission from Ref. [85]. Copyright 2017 Elsevier B.V.

Except for air quality monitoring, the inspection of the quality of water bodies in the environment is also of great significance. Recently, Noori and coworkers developed a paper-based sensor that could simultaneously detect multiple phenols in an environmental water sample within 15 min with a portable, disposable, and cost-effective novelty. Among which, in the presence of phenols, polyphenol oxidase that integrated onto the paper-catalyzed phenols, and then, the redox product would react with 3-methyl-2-benzothiazolinone hydrazine chromophore to deliver a phenol concentration-dependent color change (Figure 5a). For more attention, the polyphenol oxidase was extracted from potato peels. The long storage time (30 days) even at room temperature of this enzyme-based biosensor made it much more reliable for site investigations and nonexpert operations [34].

Despite the phenols, toxic and nonbiodegradable heavy metals are also a huge hazard that can eventually accumulate in human bodies through the bioaccumulation of the ecological system. This will lead to the heavy damage of organs or disorder of the nervous system of humans. Currently, colorimetric, fluorescent, electrochemical, and chemiluminescent paper-based sensors proved their practicability in sensing heavy metals [86]. Yu’s team designed a type of Fe^3+^-sensitive metal–organic framework nanocrystal material for fluorescence-based Fe^3+^ detection (Figure 5b) [83]. Among which, the fluorescence of the nanocrystal was quenched after the introduction of Fe^3+^ due to the interactions between Fe^3+^ and hydroxy or carboxylic acid oxygen atoms of the nanocrystal material. With this sensor, the researchers could assess the Fe^3+^ concentration in the drinking water with a low detection limit (1 μM). Chen and coworkers developed a paper-based sensor for the selective detection of Ag^+^ in human plasma, bovine serum, lake water, and tea water [35]. Interestingly, researchers proved that the sensor’s fluorescence was recovered with the additional introduction of serine after Ag^+^-induced fluorescence quenching due to the stronger binding between serine and Ag^+^ (Figure 5c).

Notably, Zhou and coworkers delivered a paper-based sensor that detected Cu^2+^ with a ratiometric property [36]. The ratio detection in that strategy was established by integrating Cu^2+^-sensitive and Cu^2+^-insensitive dyes P2017 and B001 onto the sensor. During detection, the fluorescence of P2017 decreased with the Cu^2+^ addition. In contrast, the fluorescence of B001 showed resistance to Cu^2+^ and was stably maintained. Superiorly, the ratio-based strategy significantly enhanced the signal-to-noise ratio in the background and greatly eliminated the interference of photobleaching due to the self-calibration effect. Accordingly, the sensor showed an ultra-low detection limit of 2.4 nM. Moreover, it was found that both Cu^2+^ and Hg^2+^ could interfere with the fluorescence of one single fluorophore (Figure 5d) [84]. Based on the quantum dot Cu^2+^- and Hg^2+^-sensitive fluorescence, a novel three-dimensional paper-based sensor was developed with high portability and admirable sensitivity (Figure 5e) [85]. Those paper-based sensors devoted to evaluating heavy metal ions would significantly promote environment inspection with better efficiency and convenience than space-occupied laboratory instruments concerning heavy metal pollution in vast wild fields.

## 6. Conclusions and Future Outlooks

**Figure 6:**
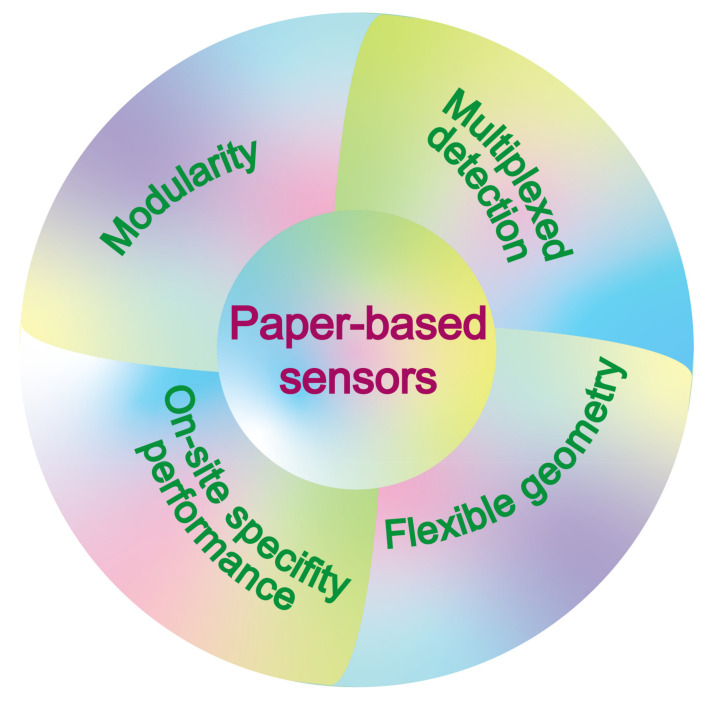
Developing directions for paper-based sensors with more practicality and promotional applications.

Paper-based devices are developed to solve the analytical situations both inside and outside of laboratory settings, mainly to perform outdoor onsite detections with high accuracy. They develop significantly due to their meager cost, excellent portability, and the same sensitivity as heavy laboratory instruments. In this review, we demonstrated the construction, working principle, and responding mechanisms of paper-based sensors from the perspective of detection analytes. Among those applications, the most striking was the specific detection of biomarkers based on the reaction between the antibody and antigen, since it provides a new means for the effective early diagnosis and pretreatment of clinical disease. Based on the abovementioned state-of-the-art demonstrations of the current paper-based sensors, although the paper-based sensors have been extensively developed in the past decades, there are insufficiencies like (1) the harsh storage conditions and short shelf times that limit their long-term storage and commercial applications, (2) the singularization of test substances does not meet the requirements of certain joint substance tests, (3) there were only detected strain pressures in one or two dimensions, and (4) when testing actual samples, the laboratory instrument’s assistance is needed, and there is a low resistance to the interfering factors in actual samples. For our consideration, solutions and developing futures of paper-based sensors were shown as follows (Figure 6).

(1) In terms of the modularity for a long shelf time and commercial application. Although the most reported paper-based sensors have relatively simple syntheses and moderate storage times, enzyme-, antibody-, and DNA template-based sensors still have complicated manufacturing processes and require sophisticated experimental operations. Moreover, their short shelf times also hamper their commercial application in rapid onsite detection. The development of modularly assembled paper-based sensors may be a solution to this situation that allows the separation of the core components to the paper matrix and signal transmission accessory parts, for which the core components with poor environmental tolerance are stored by vacuum plastic film coating and only need to be taken out when used for detection. Then, the sensor function is realized after the splicing of the activated core components and the other accessory parts.

(2) In terms of developing paper-based sensors with multiplexed detection. It is noticeable that the most current paper-based sensors perform single-analyte detection. The detection sensitivity and limit of them usually cannot meet the detection requirements for the actual sample. Furthermore, integrating different sensing materials into the same or neighboring parts of the paper matrix makes the construction of paper-based sensors more demanding and cumbersome, hindering their wide commercial applications. Attention should be paid to developing paper-based sensors with multiplexed analyte detection. The development or screening of materials and substances that respond to dual or multiple environmental factors is an inevitable trend in the future. For instance, the dual-responding paper-based sensor activates pressure, gas, or chemical analyte detection only when the environmental humidity reaches a certain level. This means conditionally activated dual-factor detection, in which swelling cellulose allows the connection of cracked conductive material layers in their dried state, forming a complete current path. This is extremely meaningful for the development of paper-based sensors to break into the field of intelligent detection. In particular, paper-based sensors that allow a higher accuracy and sensitivity even without adopting enzyme-linked or antigen–antibody-specific reactions are of great significance for high-precision analyses in enzyme- or antibody-unsuitable situations.

(3) In terms of flexible paper-based strain sensors for object–geometry-matched detection. There is indeed the fact that the most current paper-based sensors only make use of the easy modification, portability, and low-cost features of the paper matrix. Conversely, the flexible characteristic of the paper matrix is ignored, and the flexible (transformation in a multidirectional dimension) paper-based sensors developed are even more minimal. The development of paper-based sensors with object–geometry-matched property can allow the exclusion either of a sensor’s sensitivity damage or function loss caused by collision or a fold in the production, storage, or transportation procedures. Furthermore, sticking to an irregular subject with a nonplanar structure can inspire a new type of paper-based sensor that detects the strain in multiple directions. That is, it allows paper-based sensors to make a break from one-dimensional or two-dimensional pressure detection to three-dimensional holographic strain detection. At the same time, in order to adapt to the deformation performance of the flexible sensor, the development of new materials with good flexibility is also a new and challenging opportunity.

(4) In terms of onsite inspection with a high specificity performance. It should be noted that most of the current paper-based sensors require a sample pretreatment before detection, indicating their inconvenience for rapid detection in the field. Developing and screening new materials and substances with specific and susceptible responses to the analytes in a complex environment will significantly reduce unnecessary sample processing. In particular, smartphone-assisted colorimetric detection deserves to receive more attention, since it has a higher discrimination degree and accuracy than the naked eye observation and provides nonbiased detection results. Moreover, it can effectively avoid the requirement of bulky electrochemical workstations or other large instruments when performing onsite detections in the field.

Convenient and long-time storage is critical to promote broad commercial applications of paper-based sensors in the future. Additionally, multiplexed analyte detection can promote paper-based sensors to adapt to the future developing trend of multiple detections. For paper-based strain sensors, the break from two-dimensional to three-dimensional holographic detection can expand and free it from its limited multidimensional detection and provide it with huge application potential in the engineering field. Finally, considering the complexity of the sample in field detection, the specific detection of the analyte by the paper-based sensor is the key to ensuring its regular operation. There is expected to be more rapid and instructive developments of paper-based sensors through these outlooks, providing excellent references for future large-scale commercial applications.

## Figures and Tables

**Table 1:** Paper-based sensors for physical, biological, and chemical factor sensing on account of the output signal, working principle, analyte, detection limit, and shelf time.

Output Signal	Principle	Analyte	Limit	Shelf Time	Year	Ref
current	Swelling of cellulose network after absorbing water causes the separation of the conductive carbon nanotubes attached to cellulose network, increasing sensor’s resistance, then the sensor’s current changed by the humidity.	humidity	-	Three months	2020	[18]
resistance	Applied pressure reduces the gap between the conductive paper layers carrying the reduced graphene oxide material, then the sensor’s resistance decreases.	pressure	-	-	2017	[19]
resistance	Conductive molybdenum carbide-graphene layers with porous and coral-shaped microstructure on the paper matrix allow micro-cracks under external compressive or tensile pressures, which eventually causes a resistance increase of the sensor.	pressure	-	-	2020	[20]
current	Low pressure reduces the gap between the PVP layer containing conductive carbon nanotubes and the interdigital electrodes, resulting in a resistance decrease. While high pressure reduces the gap between the conductive carbon nanotubes inside the PVP layer to reduce the sensor’s resistance, leading to an increased current.	pressure	8 Pa	-	2019	[21]
resistance	Vibration caused deformation of the sensor, which increased/decreased the distance between the conductive grooves’ two walls and subsequently increase/decrease the resistance.	vibration	-	-	2021	[22]
capacitance	External pressure caused an increment of the contact area among the ionic and conductive cellulose fibers, leading to a capacitance rise of the ISP sensor.	pressure	6.25 Pa	-	2019	[23]
current	After capturing the target miRNA, PtCuMOFs/ DNA3, and PtCuMOFs/DNA4 reporter probes are hybridized to the captured miRNA on the electrode surface and generates faradaic current with conjugated methylene blue and ferrocene.	miR−141, miR−21	0.1 fM/L	-	2019	[24]
current	The hairpin structure of H1 is opened after the miRNA−155 binding. Then the hairpin structure H2 competes with miRNA−155 from H1 through a stronger combination with H1, then continuously going through this process. Then the amplified H1-H2 duplexes combine with S1-AuNPs@Cu-MOFs to oxidize glucose to generate an electrical signal.	miRNA−155	0.35 fM/L	-	2017	[25]
current	After the mutation-related DNA is captured on the sensor, it then hybridizes with another DNA strand labeled with HRP and catalyzes hydrogen peroxide to generate electrical signals.	EGFR mutation DNA	0.167 nM/L	Four weeks	2017	[12]
fluorescence	NH_3_-triggered Structure change of NH_2_-MIL-125(Ti) on the sensor is utilized for the visible fluorescence immunoassay of target CEA by coupling with a sandwich-type detection mode in the microplates.	CEA	0.041 ng/mL	-	2018	[26]
chemiluminescence	Lactate oxidase on the sensor oxidizes lactate to generate hydrogen peroxide. Subsequently, the HRP fixed on the sensor catalyzes the oxidation of TMB by hydrogen peroxide to realize the lactate-concentration-dependent color delivery.	L-lactate	0.1 mM/L	-	2017	[27]
electrochemical potential	The glucose oxidase on the sensor oxidizes glucose to generate hydrogen peroxide, which changes the redox potential of the solution directly.	glucose	0.02 mM/L	One month	2018	[28]
resistance	Adsorption of PSA through the specific reaction between PSA and PSA antibody increases the distance between the multi-wall carbon nanotubes on the sensor, forcing the resistance increment.	PSA	1.18 ng/mL	Two hours	2018	[15]
fluorescence	In the presence of the analytes, a layer-by-layer self-assembly reaction is allowed due to the recognition between the β-cyclodextrin-coated gold nanoparticles and 1-adamantane acetic acid or tetrakis (4-carboxyphenyl) porphyrin. And then, the accumulation of Au nanoparticles will give an increasing fluorescence.	carcinoembryonic antigen, p24 antigen	dozens of molecules per strip	-	2019	[29]
fluorescence	Reduced Ag^+^ in the silver nanoparticles to Ag atoms will aggregate into oligomeric clusters, distributing to the UV–vis absorption band.	AA	82.8 µM/L	Three weeks	2015	[8]
current	Adsorption of norepinephrine or serotonin gives an electrode fouling, leading to the decrease of oxidation current and the shift of the positive potential peak.	NE, 5-HT	2.5 µM/L, 0.5 µM/L	-	2017	[30]
chemiluminescence	Doxycycline relieves the expression of the reporter of the yeast bacteria, causing a noticeable color change in the sensor.	doxycycline	-	One year	2015	[16]
current	Ethanol oxidase on the senor oxides ethanol to generate hydrogen peroxide. Then the hydrogen peroxide reacted with the Prussian blue/carbon black nanoparticles to trigger an electric current on the sensor.	ethanol	0.52 mM/L	Three weeks	2017	[31]
	After the capture of the ALP by the ALP antibody on the sensor, it will catalyze the color-developing substrate to deliver an ALP-concentration-dependent color change.	ALP	0.87 U/mL	Four weeks	2019	[32]
fluorescence	In the presence of CO_2_, the fluorescent spectra peak of the P4VB on the sensor shows a CO_2_-concentration-dependent redshift.	CO_2_	5.7 ppm	-	2020	[33]
chemiluminescence	After the oxidation of phenol substrates by the polyphenol oxidase, the oxidization product will react with the benzothiazolinone hydrazine to present a color change of the sensor paper.	catechol, phenol, p-cresol, 4-methyl catechol	0.5 µM/L	One month	2020	[34]
fluorescence	Ag^+^ binding quenches the fluorescence of the CdTe quantum dots.	Ag^+^	13.16 nM/L	-	2020	[35]
fluorescence	In the presence of Cu^2+^, the fluorescence of the fluorophore P2017 decreased while that of B001 was maintained well, thus giving a ratio-based fluorescence detection technology.	Cu^2+^	2.4 nM/L	-	2019	[36]
